# sNASP and ASF1A function through both competitive and compatible modes of histone binding

**DOI:** 10.1093/nar/gkw892

**Published:** 2016-10-05

**Authors:** Andrew Bowman, Akiko Koide, Jay S. Goodman, Meaghan E. Colling, Daria Zinne, Shohei Koide, Andreas G. Ladurner

**Affiliations:** 1 Biomedical Center Munich, Physiological Chemistry, Faculty of Medicine, Ludwig-Maximilians-Universität München, Großhaderner Str. 9, 82152 Planegg-Martinsried, Germany; 2 Department of Biochemistry and Molecular Biology, University of Chicago, Chicago, IL 60637, USA; 3 Perlmutter Cancer Center, New York University Langone Medical Center, New York, NY 10016, USA; 4 Center for Integrated Protein Science Munich (CIPSM), Ludwig-Maximilians-Universität München, Butenandt Str. 5–13, 81377 Munich, Germany; 5 Munich Cluster for Systems Neurology (SyNergy), Ludwig-Maximilians-Universität München, Feodor Lynen Str. 17, 81377 Munich, Germany

## Abstract

Histone chaperones are proteins that interact with histones to regulate the thermodynamic process of nucleosome assembly. sNASP and ASF1 are conserved histone chaperones that interact with histones H3 and H4 and are found in a multi-chaperoning complex *in vivo*. Previously we identified a short peptide motif within H3 that binds to the TPR domain of sNASP with nanomolar affinity. Interestingly, this peptide motif is sequestered within the known ASF1–H3–H4 interface, raising the question of how these two proteins are found in complex together with histones when they share the same binding site. Here, we show that sNASP contains at least two additional histone interaction sites that, unlike the TPR–H3 peptide interaction, are compatible with ASF1A binding. These surfaces allow ASF1A to form a quaternary complex with both sNASP and H3–H4. Furthermore, we demonstrate that sNASP makes a specific complex with H3 on its own *in vitro*, but not with H4, suggesting that it could work upstream of ASF1A. Further, we show that sNASP and ASF1A are capable of folding an H3–H4 dimer *in vitro* under native conditions. These findings reveal a network of binding events that may promote the entry of histones H3 and H4 into the nucleosome assembly pathway.

## INTRODUCTION

Histone chaperones are defined as proteins that contribute to the thermodynamic assembly of nucleosomes without being part of the final product. Histone chaperones adopt numerous proteins folds, often containing multiple domains and can exist in multi-subunit complexes (1). Many of these interactions have been elucidated at the structural and biochemical level, revealing a highly diverse repertoire of histone interaction modules (2,3). Understanding how these interactions function together in multi-protein complexes is crucial for a better understanding of the process of nucleosome assembly.

With regards to histone chaperones specific to H3–H4, a series of crystal structures has demonstrated how this specificity is achieved, supporting the concept of ‘histone hand-off’ between chaperoning complexes proposed from previous biochemical analysis of histone chaperone interactions (2,4). For example, the unique specificity of the HIRA complex for histone H3.3 is mediated through the recognition of three residues unique to H3.3 by UBN1 (5). The binding site occupied by UBN1 is not compatible with the structure of the chaperone DAXX in complex with H3.3–H4 (6), in support of previous findings showing that the DAXX-ATRX and HIRA form two discrete complexes in the cell (7,8). Similarly, the histone chaperone ASF1 was shown through biochemical analysis to bind to histones H3–H4 concomitantly with the MCM helicase complex (9–11), a finding supported by recent crystal structures of the ASF1–H3–H4–MCM2 co-chaperoning complex (12,13). These tertiary and quaternary complexes are thought to represent snapshots of dynamic ‘histone hand-off’ mechanisms during histone folding and chromatin maturation.

The human chaperone NASP represents a unique family of TPR motif containing proteins that interact specifically with histones H3–H4 (14–16). In the cell, NASP is found in a multi-subunit complex containing the co-chaperones RbAp46 and ASF1A and the histone acetyltransferase HAT1, amongst other components (8,10,11,17–22). This interaction network is highly conserved, occurring in evolutionary distant organisms such as the budding yeast *Saccharomyces cerevisiae* (17,23) and the ciliated protozoan *Tetrahymena thermophila* (24). We recently demonstrated that the TPR domain of sNASP (the somatic splice isoform of human NASP) binds to a discrete H3 peptide motif found within the globular core of the H3–H4 dimer with nanomolar affinity (25). Interestingly, whilst the NASP epitope is distinct from the interaction site of RbAp46 and HAT1 (26–29), the interaction site overlaps significantly with that of ASF1 (30–32). This raised the question of how two histone chaperones are found in complex with each other when they share the same binding site for their histone substrate.

In order to reconcile these findings, we undertook a comprehensive interaction analysis between sNASP and ASF1A and their histone cargo. Using *in vitro* biochemical reconstitution assays we confirmed that sNASP and ASF1A do indeed compete for the C-terminal epitope of H3, with sNASP outcompeting ASF1A, and also discovered that sNASP forms a stable complex with full length H3, but not with H4. Interaction analysis with an H3–H4 dimer revealed that both sNASP and ASF1A can interact with the same dimer at the same time, and that sNASP contributes to the solubility of the hetero-tetrameric complex under physiological conditions. Importantly, using a cellular interaction assay we show that ASF1A outcompetes sNASP at the C-terminus of H3 when bound to the H3–H4 dimer, as is suggested by the crystal structure of ASF1 (30), and propose that additional interactions between sNASP and H3–H4 must exist to retain sNASP within the sNASP–H3–H4–ASF1A complex. To investigate this further we generated two site-specific monobodies against sNASP that revealed additional interaction sites involving the central acidic domain that interrupts the TPR2 motif, and one other site on the TPR domain that lies outside of the central H3 peptide-binding channel. Interestingly, these additional interaction sites were occupied both when sNASP was in complex with H3 alone and when sNASP was in complex with an H3–H4 dimer, suggesting that sNASP may hold H3 in a conformation that is conducive to folding with H4. To test this hypothesis, we carried out *in vitro* folding reactions and found that sNASP and ASF1A are capable of producing a folded H3–H4 dimer from monomeric subunits under physiological conditions, and that precomplexation of sNASP with histones before the addition of ASF1A was necessary for efficient folding to occur. Our findings reveal a dynamic interplay between two conserved histone chaperones, and suggests an intricate network of histone binding events that contribute to efficient H3–H4 folding and entry into the histone deposition pathway.

## MATERIALS AND METHODS

### Protein expression and purification

sNASP was expressed in bacteria as an N-terminal (His)_6_ fusion construct using Ni-NTA affinity resin (GE Healthcare) and was further purified by ion-exchange and gel filtration chromatography after cleavage of the (His)_6_ fusion by TEV protease, as described previously (25). sNASP mutants, truncations and the budding yeast homolog Hif1 were expressed and purified using the same method. N-terminal tagged GST-ASF1A was expressed in bacteria and purified over a Glutathione Sepharose resin (GE Healthcare), the GST tagged removed with Precision Protease cleavage, and further purified using anion exchange and gel filtration chromatography. Mb13 was expressed as a (His)_6_, Avitag fusion in bacteria, and after cleavage of the (His)_6_ fusion, was further purified by ion exchange and size exclusion chromatography. Full length *Xenopus* histones H3 and H4 were purified and refolded as described previously to form the H3–H4 dimer/tetramer (33). For reconstitution of monomeric histones with chaperones, H3 and H4 were dialyzed to water and then added directly to the chaperone. MBP–H3 (116–135) and MBP-mb1 were expressed in the same way as sNASP and purified over Dextrin Sepharose (GE Healthcare) in 20 mM HEPES–KOH pH 7.5 and 0.5 M sodium chloride, eluted in the same buffer supplemented with 10 mM maltose. Maltose was removed by dialysis prior to storage at –80°C.

### Analytical gel filtration

Analytical gel filtration was carried out using either a Superdex S200 10/300 GL column, or later a Superdex 200 Increase 10/300 GL column (GE Healthcare), in 20 mM HEPES–KOH pH 7.5 and 200 mM sodium chloride, unless otherwise stated in the text. Samples were made up in the same buffer containing 5 mM dithiothreitol prior to separation. Typically, 0.5 μl fractions were collected encompassing the void and bed volumes of the column. Fractions were separated by SDS-PAGE and stained with Coomassie Brilliant Blue (SERVA). Proteins and complexes were reconstituted at a concentration of 20 μM, unless otherwise stated in the text, before separating out by gel filtration chromatography. For sNASP N330 and sNASP cTPR, the sNASP mutants were added to equimolar amounts of ASF1A–H3–H4 in 20 mM HEPES–KOH pH 7.5 and 500 mM NaCl. The higher salt concentration was used to prevent potential precipitation of the ASF1A–H3–H4 complex in the absence of sNASP binding. Gel filtration was then carried out as above in 20 mM HEPES–KOH pH 7.5 and 200 mM sodium chloride.

### Solubilization of ASF1A-H3–H4 through salt titration, or by sNASP

For the salt titration, ASF1A was mixed with H3–H4 dimers at 20 μM each in a volume of 50 μl containing 20 mM HEPES–KOH pH 7.5 and 0.2 M sodium chloride, resulting in near complete precipitation of the complex. Sodium chloride was then titrated to the concentrations shown in Figure 2A. Samples were incubated for 30 min at 30°C, before separating soluble and precipitated material by centrifugation. The precipitate was lightly washed with 50 μl of the same buffer to remove residual soluble protein. Insoluble proteins were solubilized by addition of 2× SDS-PAGE loading buffer and equal volumes of insoluble and soluble material were separated by SDS-PAGE. For the sNASP titration (Figure 2B), ASF1A and H3–H4 were made up as before, but increasing concentrations of sNASP were titrated into the precipitate whilst keeping the sodium chloride concentration constant.

### Fluorescence-2-hybrid assay

For analysis of protein–protein interactions using the F2H assay we used a human U2OS cell line harbouring the stably integrated LacO (256×) array, as has been described earlier (34). F2H assays were essentially performed as previously described in (35,36) and (25,34). U2OS cells were cultured in DMEM containing 10% FBS (GIBCO), 2 mM l-glutamine (Sigma), 100 U ml^−1^ penicillin, 100 μg ml^−1^ streptomycin (Sigma) in a humidified atmosphere with 5% CO_2_ at 37°C. Transient transfections of cells seeded onto an eight-well μ-Slide (ibidi) were carried out using Xfect (Clonetech) according to manufacturers instructions. Imaging was carried out on a Zeiss AxioObserver Z1 confocal spinning disk microscope equipped with an AxioCam HRm CCD camera (Zeiss) through a Zeiss C-Apochromat 40×/1.2 water immersion objective lens. Image analysis was manually performed with ImageJ image analysis software. Fluorescence intensity at the LacI array was calculated as a percentage increase over the nucleoplasm. Briefly, a region of interest (ROI) was drawn around the LacO array as demarcated by the LacI-mCherry fusion protein. The average intensity of the fluorescence from the ROI was then compared to the average intensity from a same size ROI taken from an adjacent region of the nucleus. The Wilcoxen Rank Sum Test was used as a normal distribution could not be assumed. Only cells in which a clearly discernable array was present were counted. As with previous experiments (25), a total of 20 cells were counted as not to inflate the calculated *P*-value.

### Selection of monobodies and affinity measurements

A phage-display library of monobodies, dubbed ‘side libarry’ (37) was used for the selection of the sNASP-binding monobodies using previously described methods (37–39). A total of four rounds of phage-display library selection were performed using the target concentrations of 100, 100, 50 and 50 nM. The enriched pools of monobody clones were converted into yeast libraries after performing gene shuffling among them, as described previously (37), and two rounds of library sorting using fluorescence-activated cell sorters were performed using the target concentrations of 500 and 250 nM. Affinity of the monobodies was measured using the yeast display method as described previously (39). We have validated using numerous monobodies that the *K*_D_ values measured in this manner are consistent with those determined from biophysical methods using purified monobodies (39–41). Cell lysate of HEK293T cells was prepared in the high salt buffer (10 mM Tris–HCl pH 8, 420 mM NaCl, 0.1% NP-40) and the lysate concentration was adjusted so that its OD_280nm_ in the binding assay was 3.0.

### Analysis of monobody–sNASP interactions

Analytical gel filtration of sNASP complexes with monobodies was carried out under 200 mM NaCl, 20 mM HEPES–KOH pH 7.5 and 1 mM EDTA on a Superdex 200 10/300 GL column (GE Healthcare). 30 uM of each component was made up in the same buffer with 2 mM DTT, with monobodies being added first followed by sNASP, histones and then ASF1A. Peaks from the elution profiles were separated out on SDS-PAGE gel and stained with Coomassie Brilliant Blue (SERVA).

### H3–H4 folding assay

Purified sNASP and ASF1A were dialyzed to 200 mM sodium chloride, 20 mM HEPES–KOH pH 7.5, concentrated to 200 μM and supplemented with 2 mM dithiothreitol. Lyophilized H3 and H4 were dissolved in water at a concentration of 200 μM and supplemented with 2 mM dithiothreitol. 5 μl of each component was added to 30 μl of buffer containing 200 mM sodium chloride and 20 mM HEPES–KOH pH 7.5, in all possible orders of addition, to give a final volume of 50 μl and final concentration of 20 μM. Samples were incubated at 30°C for 30 min before isolating soluble and insoluble material by centrifugation. Proteins were separated by SDS-PAGE and stained with Coomassie Brilliant Blue (SERVA). Total relative precipitation from each lane was quantified using ImageJ (http://imagej.net/Fiji). The maximum and minimum values were used to normalise values from three independent experiments.

### H3–H4 deposition assay

Deposition of H3–H4 onto DNA was carried out in a similar fashion as has been previously reported (42–45). A 91 base pair DNA corresponding to the central portion of the Widom 601 DNA sequence (46,47) was mixed at a final concentration of 500 nM with 1, 2 or 4 μM of prefolded H3–H4 dimers, unfolded H3 and H4, sNASP–H3–H4–ASF1A complex made from prefolded histones, or the same complex made from unfolded histones, in a final volume of 20 μl. The buffer composition was 20 mM HEPES–KOH pH 7.5 and 200 mM NaCl. Samples were incubated at 25°C for 1 h, precipitates removed by centrifugation, 1 μl of soluble material was added to 10 μl of 4% sucrose and resolved on a 7% poly-acrylamide gel in 0.5% TBE buffer at 4°C. The gel was visualized by staining with ethidium bromide.

## RESULTS

### sNASP and ASF1A bind competitively to both an H3 C-terminal peptide and to full length H3

**Figure 1.**
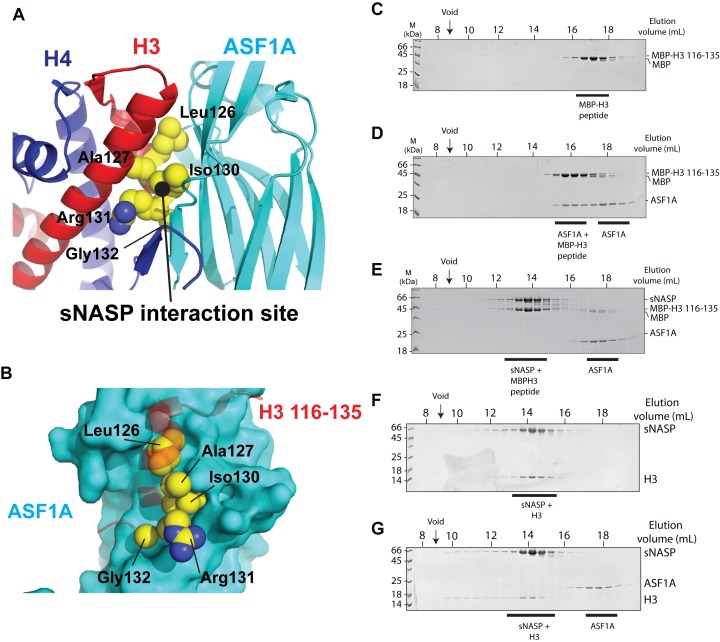
sNASP and ASF1A bind competitively to histone H3. (**A**) Crystal structure of ASF1A bound to an H3–H4 dimer (PDB code: 2IO5) with residues from the previously mapped sNASP binding site (25) shown as yellow spheres. (**B**) Detailed view of H3 residues involved in binding the TPR domain of sNASP in complex with ASF1A. (**C**) Gel filtration elution profile of free MBP H3 (116–135). (**D**) Gel filtration elution profile of ASF1A bound to MBP H3 116–135 (ASF1A was kept at a molar excess over the MBP H3 (116–135) peptide to visualise both free and bound ASF1A). (**E**) Elution profile of equimolar amounts of sNASP, ASF1A and MBP H3 116–135, revealing that sNASP can effectively outcompete ASF1A for binding to the H3 C-terminal peptide. (**F**) Elution profile of sNASP complexed with full-length histone H3. (**G**) Elution profile of sNASP, ASF1A and full-length H3. ASF1A is unable to bind to H3 whilst it is associated with sNASP, eluting in its unbound fraction.

In a screen looking for the peptide binding epitope of the TPR domain of sNASP, we previously identified a short motif close to the C-terminus of H3 that bound with high affinity and specificity (25). Interestingly, residues crucial to this interaction are sequestered in the interface between H3 and the co-chaperone ASF1 (Figure 1A and B) (30–32). We therefore wanted to determine if sNASP and ASF1A bind to this region competitively. Incubation of ASF1A with an H3 peptide (incorporating residues 116–135) fused to MBP resulted in co-elution of the two proteins in a single complex as seen by coomassie staining of fractions from gel filtration chromatography (Figure 1C and D), as was previously seen with a similar H3 peptide (31). This is shown by the shifting of the MBP–H3 116–135 and ASF1A peak to earlier eluting fractions when compared to their elution alone (for ASF1A elution see Figure 1E and G). We next wanted to know whether sNASP effectively competed with ASF1A for H3 peptide binding. Mixing equimolar concentrations of MBP–H3 (116–135), ASF1A and sNASP and separating out the complexes by gel filtration chromatography, we found that the MBP–H3 (116–135) peptide co-eluted with sNASP rather than ASF1A (Figure 1E), suggesting that with respect to the very C-terminus of H3, sNASP and ASF1A bind competitively, with sNASP outcompeting ASF1A.

Next, we wanted to know whether sNASP and ASF1A binding are mutually exclusive with regard to the full-length H3 protein. Histones are notoriously aggregation-prone in the absence of their folding partner. We found, however, that sNASP formed a stable, soluble complex with H3 in the absence of H4 (Figure 1F). Interestingly, whilst soluble on their own, H3 and ASF1A formed a precipitate when mixed in stoichiometric amounts (Supplementary Figure S1A). Crucially, addition of sNASP to this precipitate resulted in solubilization of both proteins, with sNASP and H3 coeluting in a single complex and ASF1A eluting on its own when analyzed by gel filtration chromatography (Figure 1G). In addition to H3, we also tested for potential interaction between sNASP and histone H4. We found that while remaining soluble, the majority of histone H4 formed a high molecular weight aggregate with sNASP, which eluted in the void volume of the column (Supplementary Figure S1B). These experiments demonstrate that ASF1A and sNASP compete for binding to the C-terminal region of H3, in agreement with previous structural and biochemical analysis of these chaperones (25,30), and that sNASP forms a soluble complex with full length H3 that excludes ASF1A binding.

### sNASP and ASF1A bind compatibly to an H3–H4 dimer

**Figure 2.**
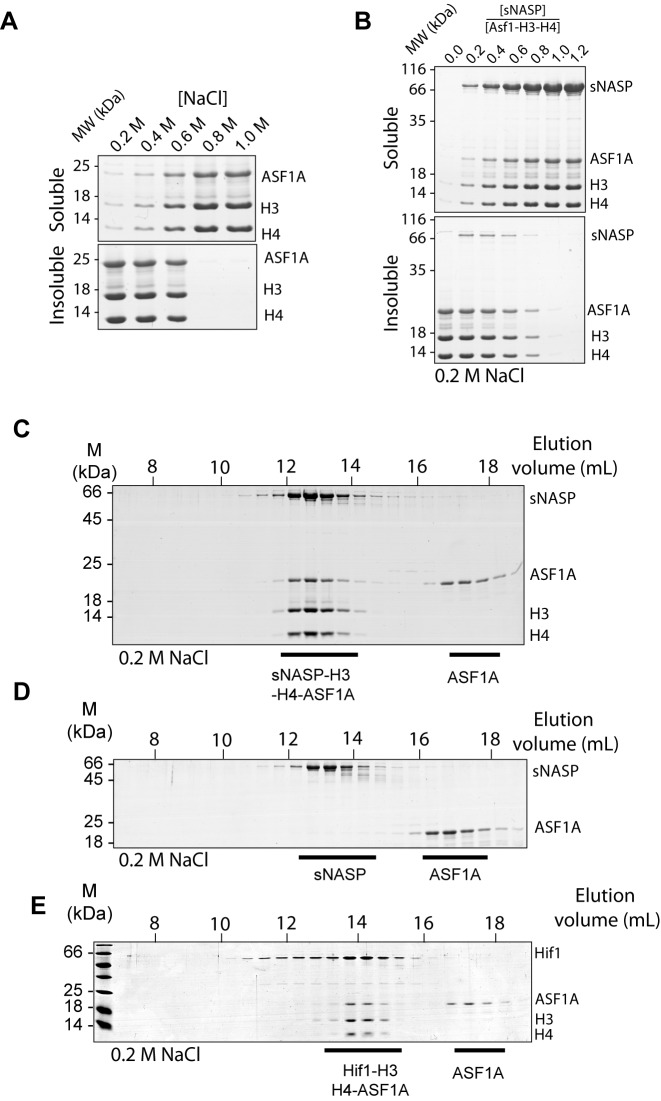
sNASP solubilises H3–H4–ASF1A by forming a stable sNASP–H3–H4–ASF1A complex. (**A**) Salt titration showing the solubility of the H3–H4–ASF1A complex is dependent on ionic strength. Soluble and insoluble material were separated by centrifugation before analysis by SDS-PAGE PAGE and coomassie staining. (**B**) The precipitate formed at lower ionic strength conditions in (A) (200 mM sodium chloride) can be solubilized through titration of sNASP. At an equimolar ratio of sNASP to H3–H4–ASF1A near complete solubilization is observed. (**C**) Gel filtration elution profile of sNASP bound to the H3–H4–ASF1A complex showing co-elution of all four proteins as visualized by SDS-PAGE and coomassie staining. A molar excess of ASF1A over all other components was used to gauge the stoichiometry of the complex. (**D**) Gel filtration elution profile of sNASP and ASF1A showing that the two chaperones elute in separate fractions, and therefore do not interact in the absence of their histone cargo. (**E**) Elution profile of the yeast homolog of sNASP, Hif1, showing that the complex formed between sNASP family of histone chaperones and H3–H4–ASF1A is evolutionary conserved.

Although a complex containing sNASP and ASF1A has been extensively studied previously, the majority of these studies have focused on chaperoning complexes isolated from cultured cells, where a number of other components were present (8,11,15–21). To determine if sNASP and ASF1A can form a complex with H3–H4 in the absence of other factors, we attempted an *in vitro* reconstitution using purified chaperones and an H3–H4 dimer. As the complex formed between ASF1A and H3–H4 is prone to precipitation under physiological salt concentrations (30,32,48) (Figure 2A), we initially attempted to reconstitute complexes in high salt buffers (0.6 M sodium chloride or higher). This attempt failed, as sNASP did not associate with ASF1–H3–H4 under the conditions needed for ASF1–H3–H4 solubility. Interestingly, however, we noticed that titrating sNASP into a precipitated ASF1–H3–H4 complex resulted in the same solubilizing effect as raising the ionic strength (Figure 2B), similar to the effect seen with ASF1A and H3 (Supplementary Figure S1A). However, whilst ASF1A was excluded from the sNASP–H3 dimer (Figure 1G), upon separation of the solubilized precipitate by gel filtration, we observed co-elution of all four proteins, forming a sNASP–H3–H4–ASF1A tetramer (Figure 2C). We could not detect any interaction between sNASP and ASF1A in the absence of H3 and H4 under the same conditions (Figure 2D), suggesting that the interaction between sNASP and ASF1A is mediated through their histone substrate.

Previously, we were unable to detect an interaction between the budding yeast homolog of sNASP, Hif1 and an H3 116–135 peptide of H3 (25), even though the interaction was detectable in a more evolutionary distant plant homolog. We therefore wondered whether Hif1 was capable of interacting with H3–H4 whilst in complex with ASF1, as has been shown previously (23). To test this, we reconstituted the Hif1–H3–H4–ASF1A complex, and separated out the components using gel filtration chromatography. As the globular domain of human ASF1A and yeast Asf1, the main interaction site between the histone chaperone and H3–H4, is highly conserved we used the human ASF1A in these experiments. However, it should be noted that the yeast Asf1 has a longer acidic tail region compared to human ASF1A, that may carry functional importance. Interestingly, Hif1 coeluted with H3, H4 and ASF1A (Figure 2E), suggesting that although Hif1 is unable to interact with the H3 C-terminal peptide with any significant affinity (25), it has retained its ability to interact with an H3–H4 dimer in the presence of ASF1A, as has recently been suggested (49). These findings reveal that sNASP plays a crucial role in preventing aggregation of the H3–H4–ASF1A complex, and recapitulates previous *in vivo* findings showing that sNASP forms a stable complex with ASF1 and histones through interactions that are evolutionary conserved from yeast to humans.

### The TPR–H3 peptide interaction is not required for sNASP–H3–H4–ASF1A complex formation

**Figure 3.**
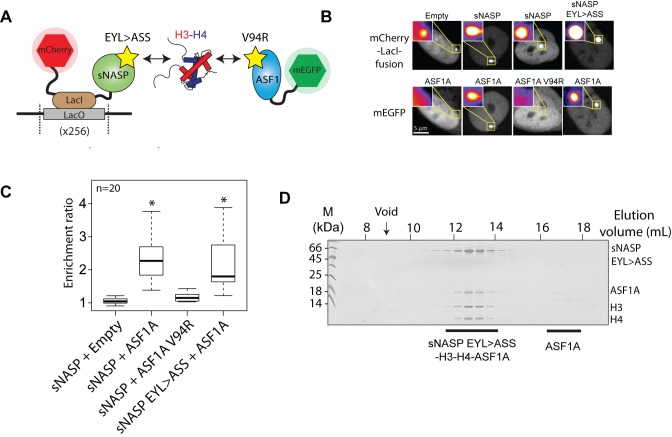
The effect of sNASP and ASF1A mutants on sNASP–H3–H4–ASF1A complex formation. (**A**) Diagrammatic representation of the F2H experiment. sNASP is tethered to a LacO array through an mCherry-LacI fusion, whilst ASF1A is expressed as a soluble mEGFP fusion. As the chaperones do not interact directly, interaction between the two chaperones is likely mediated through endogenous cellular H3–H4. Yellow stars represent mutations that disrupt the known histone binding surfaces of the two chaperones. (**B**) mEGFP-ASF1A does not recruit to the empty mCherry-LacI construct, but does recruit to the sNASP fusion. Disruption of the H3 binding interface of ASF1A by the V94R mutation abrogates recruitment of ASF1A, however, disruption of the sNASP TPR-H3 peptide interaction through the EYL>ASS triple mutation has little effect of ASF1A recruitment. (**C**) Quantification of images shown in (B). Asterisks represent a *P* value of <0.001 as determined by the Wilcoxon rank sum test. (**D**) Reconstitution of the sNASP EYL>ASS-H3–H4–ASF1A *in vitro* demonstrates that disruption of the TPR–H3 peptide interaction has little effect on the sNASP's ability to form a complex with H3–H4–ASF1A.

To reconcile the findings that sNASP and ASF1A bind compatibly to an H3–H4 dimer whilst competing for the same binding site on H3, we pursued the possibility that either sNASP or ASF1A contain a secondary histone-binding site in addition to those that have already been described. To determine which chaperone may contain the putative secondary mode of binding, we used previously defined point mutants of ASF1A (V94R) (31) and sNASP (E246A/Y249S/L253S, termed EYL>ASS) (25) that disrupt their known binding sites to histones: ASF1A V94R mutates a key hydrophobic patch that interacts with the H3 C-terminal region, whereas sNASP EYL>ASS mutates residues within the H3 peptide-binding pocket formed by the TPR repeat motifs. We hypothesized that a retained interaction in the presence of a mutation would be indicative of a secondary mode of binding. As the sub-components of the sNASP–H3–H4–ASF1A complex are prone to aggregation *in vitro* (Figure 2A) we initially employed the Fluorescent 2-Hybrid (F2H) assay to probe the complex in live cells. In the F2H assay a protein–protein interaction can be observed by assessing the ability of a bait protein, tethered to an integrated LacO array, to recruit a soluble prey protein fused to a fluorescent protein (Figure 3A) (35,36). Using this assay we found that an mCherry-LacI-sNASP bait construct efficiently recruited a soluble mEGFP-ASF1A fusion construct to the LacO array, but that mEGFP-ASF1A was not recruited to the empty mCherry-LacI fusion (Figure 3A–C). As sNASP and ASF1A do not interact directly *in vitro* (Figure 2D), this interaction is most likely mediated through endogenous H3–H4 present in the cell (as illustrated in Figure 3A). Using the ASF1A V94R mutation to disrupt histone binding, we abolished the recruitment of ASF1A V94R to the sNASP-tethered array, whereas mutation of the sNASP TPR–H3 peptide binding interface had little effect on recruitment (Figure 3B and C), suggesting that a secondary mode of binding exists between sNASP and the H3–H4 dimer. These results are in agreement with a previous study in which the V94R mutation of ASF1A failed to pull down NASP from whole cell extracts (18). To further test the idea of a secondary mode of interaction between sNASP and histones, and to exclude possible effects from extraneous cellular components, we reconstituted the sNASP–H3–H4–ASF1A complex *in vitro* using the sNASP EYL>ASS mutant and found that it forms a complex with H3–H4–ASF1A similar to wild-type sNASP (Figure 3D). Taken together, these findings suggest that it is the sNASP component of the sNASP–H3–H4–ASF1A complex which contains a secondary, as yet uncharacterized, mode of histone interaction, and that, unlike the TPR–H3 peptide interaction (Figure 1E and F), this mode of interaction is compatible with co-binding of ASF1A.

### Generation of monobodies specific to sNASP

**Figure 4.**
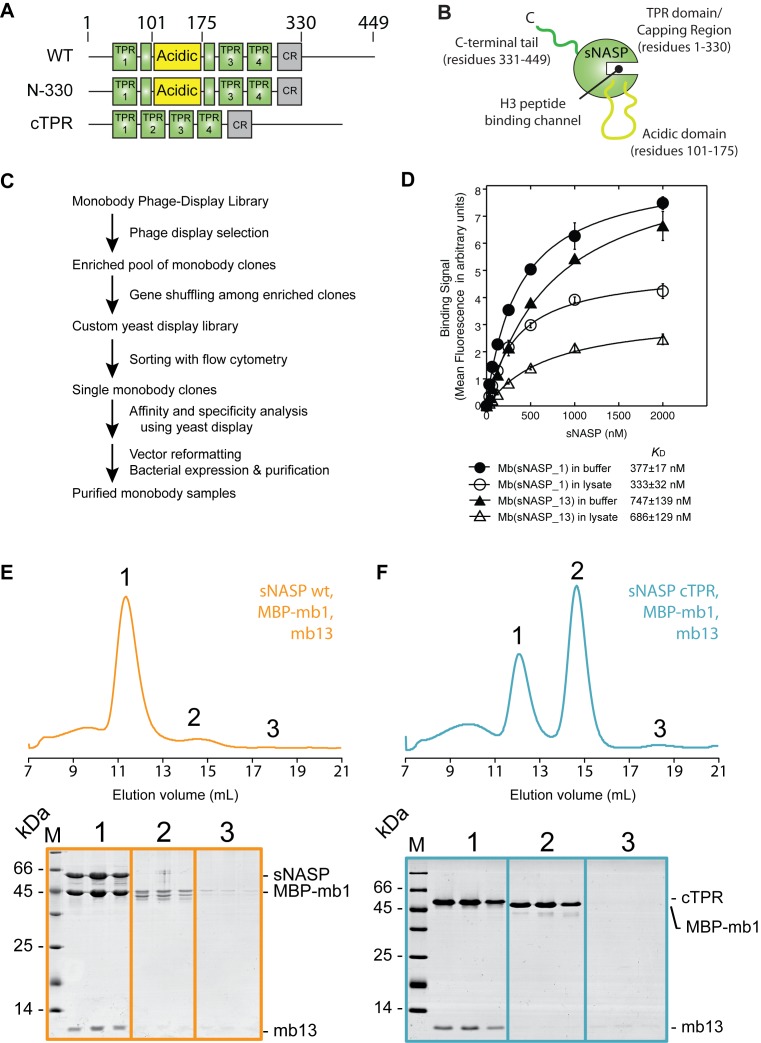
Isolation and characterization of two sNASP specific monobodies. (**A**) Domain diagrams of wild-type (WT) sNASP and truncation mutants N-330 and cTPR used in this study showing the TPR motifs in green, the acidic region in yellow and the capping region (CR) in gray. The contiguous TPR mutant (cTPR) is a deletion of the interrupting acidic domain and seamless stitching together of the TPR2 motif. The sNASP N-330 truncation was used as an antigen for monobody library screening. (**B**) Diagrammatic representation of the sNASP protein with corresponding residues shown for each domain. (**C**) Outline of monobody generation strategy. Adopted from Figure 1A of (40). (**D**) Dissociation constants of two sNASP specific binders, mbsNASP_1 and mbsNASP_13 (denoted mb1 and mb13 for brevity), were determined by yeast display. Measurements were also carried out in the presence of HEK293 lysate to probe the specificity of the interaction. The error bars on each data point show SD. from triplicate measurements. The dissociation constant (*K*_D_) values shown are the average of *K*_D_ values determined from triplicate measurements, and the errors shown are the SD. (**E**) Gel filtration analysis of MBP-mb1 and mb13 binding to full-length sNASP. Both monobodies eluted with sNASP in a single complex (peak 1), demonstrating their non-overlapping binding sites. (**F**) Gel filtration analysis of MBP-mb1 and mb13 binding to the sNASP cTPR truncation mutant. Whilst mb13 retained its interaction with sNASP in the absence of the central acidic domain (peak 1), MBP-mb13 eluted in its separate fraction (peak 2).

As a means to investigate the secondary mode of sNASP's interaction with H3–H4 further, we attempted to isolate monobody binders to sNASP (Figure 4A–C). Monobodies are synthetic, monoclonal binding proteins constructed with the fibronectin type III scaffold that can be isolated against specific antigens using various display technologies (37,50,51). They function as exquisite molecular probes in studying protein–protein interactions (40,41,52,53). In designing the sNASP antigen for monobody generation, we removed the unstructured C-terminal region of sNASP (residues 331–449), but retained the central acidic domain that interrupts its TPR2 motif so as not to overtly constrain the folded core of the TPR domain (Figure 4A and B), because we were interested in obtaining monobodies directed to the structured regions of sNASP. The resulting sNASP N-330 construct retained its interaction with the H3–H4–ASF1A complex (Supplementary Figure S2A), and was used to screen a large monobody library to isolate sNASP-specific binders (Figure 4C).

We identified two sNASP-specific monobodies termed Mb(sNASP_1) and mbsNASP_13 (for brevity referred to as mb1 and mb13; see Supplementary Figure S2B for sequence information) that interacted with sNASP with nanomolar affinity (Figure 4D), as measured by yeast display (37). Binding of mb1 and mb13 to sNASP was further tested using full-length sNASP and monobodies expressed and purified from bacteria. Upon recombinant expression, mb1 had limited solubility on its own, and so was expressed as a maltose binding protein (MBP) fusion, which also aided in resolving the two monobodies during SDS-PAGE. As mb1 and mb13 were isolated separately against a truncated form of sNASP, we first addressed whether the two monobodies interacted with full-length sNASP, and whether their binding was mutually exclusive (representing overlapping binding sites) or not (representing non-overlapping binding sites). Mixing equimolar concentrations of sNASP, MBP-mb1 and mb13 we observed that all three proteins eluted in a single peak during gel filtration chromatography (Figure 4E, peak 1), confirming their interaction with full-length sNASP, and demonstrating that they bind to non-overlapping surfaces of sNASP. Next, we wanted to know if we could further define the interaction sites of the monobodies on the surface of sNASP. Previously, we demonstrated that the central acidic domain of sNASP could be removed, leaving a contiguous TPR (cTPR) construct that retained H3-peptide binding ability (25). To test whether the acidic domain was required for either mb1 or mb13 binding, we repeated the binding experiment using the cTPR mutant. Interestingly, whilst mb13 binding was not affected, co-eluting with sNASP (Figure 4F, peak 1), MBP-mb1 eluted on its own in a separate fraction (Figure 4F, peak 2). This suggests that the acidic domain of sNASP comprises at least part of the binding epitope of mb1, whereas mb13 binds to another site on sNASP's TPR domain. Therefore, these monobodies represent unique probes for investigating the involvement of different surfaces of sNASP in histone recognition.

### Surface mapping of sNASP reveal two additional histone interaction sites required for sNASP–H3–H4–ASF1A complex formation

**Figure 5.**
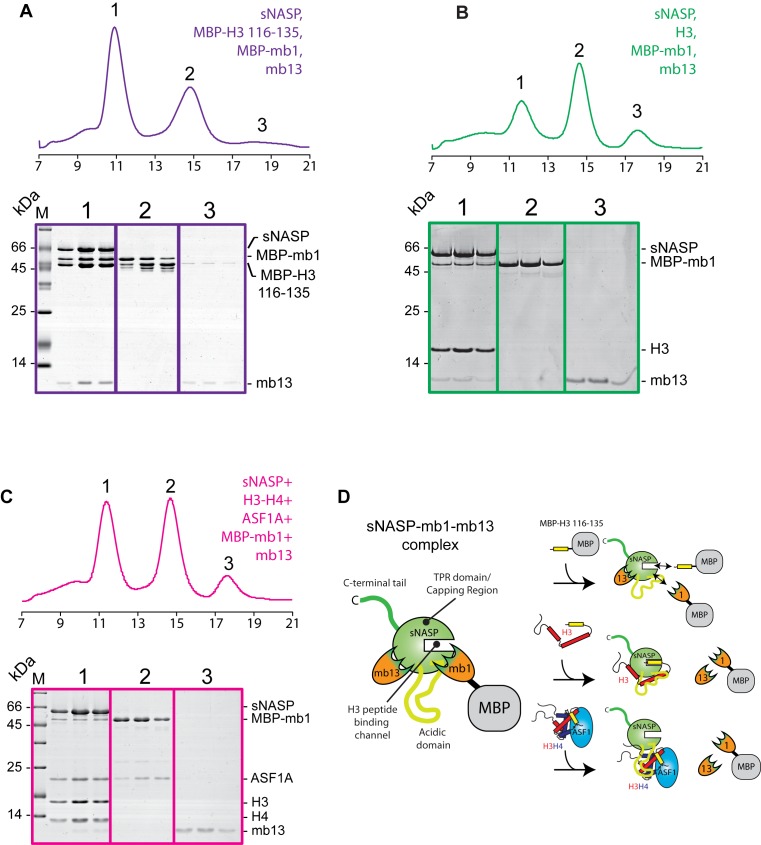
Probing the secondary modes of sNASP interaction using monobodies mb1 and mb13. (**A**) Gel filtration profile of equimolar amounts of sNASP, MBP–H3 116–135, MBP-mb1 and mb13. Whilst mb13 remains predominantly associated with the sNASP-MBP–H3 116–135 complex (peak 1), MBP-mb1 is partially displaced, suggesting that it undergoes binding site competition with the H3 peptide (free MBP–H3 116–135 and MBP-mb1 have partially overlapping elution profile represented by peak 2). (**B**) Gel filtration profile of sNASP, MBP-mb1, H3 full-length and mb13, showing that MBP-mb1 and mb13 are largely excluded from the sNASP–H3 complex (peak 1), eluting in their separate fractions (peaks 2 and 3, respectively). (**C**) Elution profile of sNASP, MBP-mb1, mb13, H3–H4 and ASF1A, showing that MBP-mb1 (peak 2) and mb13 (peak 3) are largely displaced from the sNASP–H3–H4–ASF1A complex (peak 1). (**D**) Diagrammatic representation of the complexes formed between sNASP, its various histone substrates and the monobodies mb1 and mb13. Whilst mb13 retains its binding to sNASP in the presence of the H3 116–135 peptide, it is displaced upon either H3 full-length or H3–H4–ASF1A complex binding. MBP-mb1 is partially displaced by the H3 116–135 peptide, suggesting binding site competition, and is fully displaced upon H3 full-length or H3–H4–ASF1A complex binding.

As monobodies bind to discrete surface epitopes on their target protein, we wanted to address whether the interaction sites of mb1 and mb13 overlapped with any of the histone binding interfaces of sNASP. We first tested compatibility of monobody binding with the H3 C-terminal peptide (Figure 5A). Whilst mb13 remained largely associated to sNASP in the presence of MBP–H3 (116–135), a significant proportion of MBP-mb1 was displaced, eluting in a separate peak (Figure 5A, peak 2). This suggests that mb1 and H3 (116–135) have at least partially overlapping binding sites, whereas mb13 binds to sNASP on the TPR domain/capping region (Figure 4F), but outside of the central H3 peptide-binding channel. We next tested whether binding of mb1 and mb13 is compatible with full-length H3 interaction (Figure 5B). Surprisingly, although the epitope of mb13 was outside of the H3 peptide-binding region, mb13 was displaced from sNASP upon interaction with H3. Mb1 was also displaced as expected, with the majority of both monobodies eluting in their own fractions (peaks 2 and 3), whilst sNASP and H3 eluted as a single complex (Peak 3) (Figure 5B). Finally, we addressed whether mb1 and mb13 remain associated to sNASP whilst it is in complex with H3–H4 and ASF1A (Figure 5C). Similar to full-length H3, mb1 and mb13 were displaced upon binding of the H3–H4–ASF1A complex, eluting in their individual fractions (peaks 2 and 3) when analyzed by gel filtration chromatography, whereas sNASP eluted as a complex with H3–H4–ASF1A (Peak 1) (Figure 5C). Considering the mapping of the monobody binding sites to two discrete epitopes on the surface of sNASP (Figure 4E and F), these findings suggest that sNASP makes extensive interactions with both full-length H3 on its own, and with an H3–H4 dimer in the context of the H3–H4–ASF1A complex. This secondary mode of interaction involves at least two additional interactions sites on sNASP: the acidic domain that interrupts the two helices of the TPR2 motif, and the surface of the TPR domain or capping region outside of the central peptide-binding cavity. In summary, and in support of biochemical analysis of the sNASP–H3–H4–ASF1A complex, surface mapping of sNASP using our monobodies mb1 and mb13 has revealed that sNASP makes an extensive interaction interface with both full-length H3 and with the H3–H4–ASF1A complex, and that the outer surface of TPR domain/capping region and the central acidic domain constitute at least part of this interface (for a summary of the monobody interactions see Figure 5D).

### The acidic domain of sNASP is crucial for sNASP–H3–H4–ASF1A complex formation

**Figure 6.**
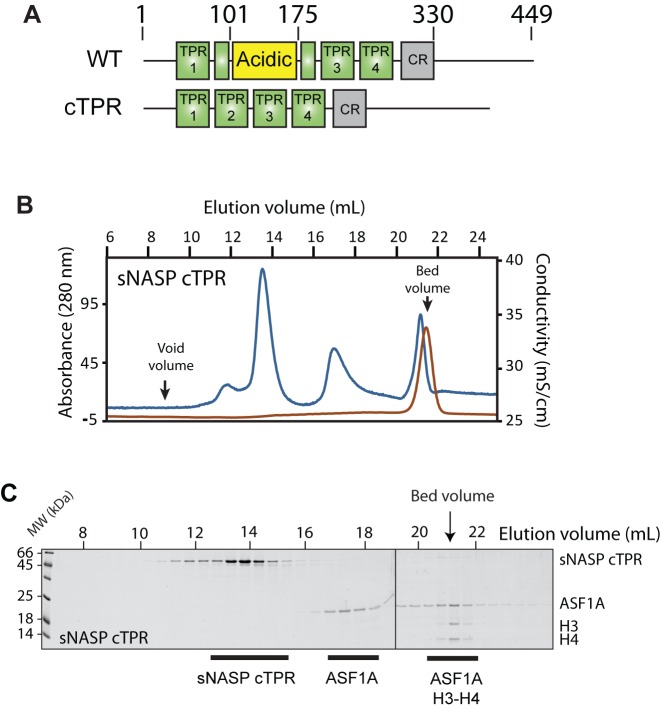
The central acidic domain of sNASP is necessary for H3–H4–ASF1A binding. (**A**) Domain diagrams of the contiguous (cTPR) truncation mutant compared to wild-type sNASP. (**B**) Elution profile of the sNASP cTPR mutant and H3–H4–ASF1A separated out by gel filtration chromatography. The conductivity spike representing the higher salt from sample preparation eluting in the bed volume of the column is indicated. (**C**) Fractions from the elution separated by SDS-PAGE reveal that the cTPR mutant, although retaining its ability to interact with the H3 116–135 peptide, is unable to bind to the H3–H4–ASF1A complex.

Next, we wanted to independently confirm the importance of the secondary histone binding sites of sNASP identified by monobody surface mapping. Attempts to gain high-resolution structural information on the mb1 and mb13-sNASP complexes through co-crystalization have thus far not succeeded, making the exact location of monobody binding difficult to determine. However, as the mb1 binding site can be disrupted by removing the acidic domain to form a contiguous TPR domain (sNASP cTPR) (Figure 4F) (25), and as mb1 is effectively displaced from sNASP by H3–H4–ASF1A, we deduced that the acidic domain may represent at least part of the sNASP binding interface with H3–H4–ASF1A. Interestingly, whilst the position and overall acidic nature of the domain is conserved amongst diverse species, we could not identify any overriding sequence conservation outside of its enrichment in glutamate and aspartate residues (Supplementary Figure S3). To further investigate the role of the acidic domain in ASF1–H3–H4 binding, we mixed the cTPR mutant with H3–H4 and ASF1A at high salt concentration (0.6 M sodium chloride) to prevent precipitation of the components (Figure 2A and B) and separated out the formed complexes by gel filtration chromatography (Figure 6A–C: ASF1A was kept at a molar excess over the other components to judge the stoichiometry of the complex and to serve as an internal control in marking the elution point of free ASF1A). Interestingly, the sNASP cTPR mutant failed to co-migrate with the H3–H4–ASF1A complex, whereas the H3–H4–ASF1A complex eluted in the bed volume of the column (Figure 6B and C). As the H3–H4–ASF1A complex is aggregation-prone under physiologically relevant salt concentrations used in the experiment (Figure 2A), elution in the bed volume most likely represents the complex being kept in solution by the higher salt concentration used in sample preparation. It should be noted that the same conditions were used for successfully separating the entire sNASP–H3–H4–ASF1A complex containing the sNASP N-330 truncation mutant (Supplementary Figure S2A), showing that under these condition of high salt loading the sNASP–H3–H4–ASF1A complex is still able to form normally. This finding suggests that the acidic domain, although dispensable for binding the H3 C-terminal peptide of H3 (25), is necessary for the secondary mode of interaction between sNASP and the H3–H4 dimer whilst it is in complex with ASF1A, supporting our view that sNASP utilizes secondary histone-binding sites.

### sNASP and ASF1 cooperate to fold an H3–H4 dimer under native conditions *in vitro*

**Figure 7.**
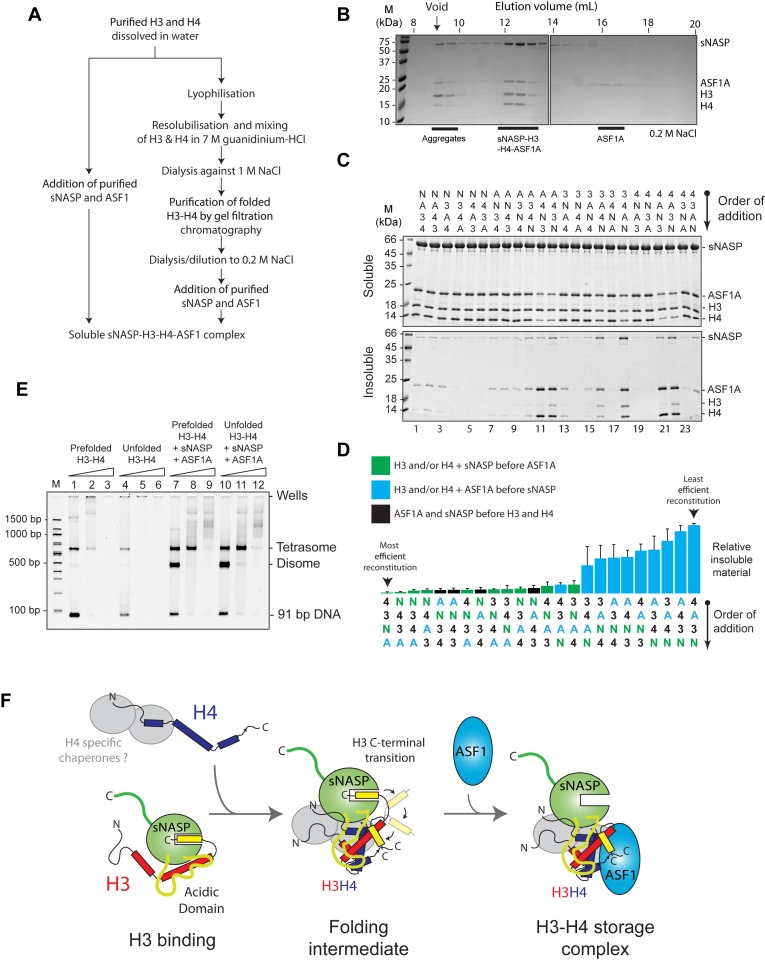
sNASP and ASF1A are capable of folding an H3–H4 dimer *in vitro*. (**A**) Flow chart showing the two different strategies for reconstituting the sNASP–H3–H4–ASF1A complex *in vitro*. (**B**) H3 and H4 monomers dissolved in water were mixed with sNASP and ASF1A and the resulting complexes separated by gel filtration chromatography. The positions of aggregates, sNASP–H3–H4–ASF1A complex and free ASF1A are shown. (**C**) Reconstituting the sNASP–H3–H4–ASF1A complex. sNASP (N), ASF1A (A), H3 (3) and H4(4) were combined in all possible orders of addition and the soluble and insoluble fractions isolated and analyzed by SDS-PAGE. (**D**) Quantification and ranking of the total relative precipitate (insoluble fraction) formed from each order of addition shown in (C). The experiment was carried out in triplicate with the error representing the standard deviation. (**E**) Tetrasome assembly assay comparing the sNASP–H3–H4–ASF1A complexes formed from either prefolded histones or unfolded histones. Positions of tetrasomes, disomes and free DNA are shown. 0.5 μM of DNA was combined with 1, 2 or 4 μM of H3 & H4 (lanes 1–3), H3–H4 dimers (lanes 4–6) or sNASP–H3–H4–ASF1A complex made from prefolded H3–H4 (lanes 7–9) or unfolded H3 & H4 (lanes 10–12), representing a 1:1, 1:2 and 1:4 molar ratio of DNA to H3–H4 tetramer in each case. (**F**) A molecular model for the role of sNASP and ASF1 in H3–H4 chaperoning. The interaction between sNASP and H3 is mediated by the TPR domain binding to the H3 C-terminus, and through additional contacts involving the acidic domain and an interaction site on the TPR domain/capping region that lies outside of the central H3 peptide-binding channel. ASF1A cannot compete for H3 binding when it is bound by sNASP. Folding with H4 causes a conformational change in H3, which results in a transition of the H3 C-terminal region from the TPR domain to its position within the globular core of the histone fold. As ASF1 recognises the folded surface of an H3–H4 dimer, this transition is accompanied by ASF1 binding at the C-terminal region of H3. sNASP is retained within the H3–H4–ASF1 complex through its secondary modes of interaction with the H3–H4 dimer that are compatible with ASF1 binding. In complex with H3–H4–ASF1, sNASP contributes to the solubility of the histones and prevents their aggregation.

As sNASP forms a stable complex with H3 in the absence of H4 (Figure 1F), sNASP may work upstream of ASF1A in the histone chaperoning pathway, scaffolding free H3 and aiding in its folding with H4 to form an sNASP–H3–H4–ASF1A complex. If this were the case, we wondered whether the sNASP–H3–H4–ASF1A complex could be reconstituted using histone monomers as substrates, bypassing the requirement for using pre-folded H3–H4 dimers (Figure 7A). To test this, we mixed sNASP with monomeric H3 and H4 (dissolved in water) and ASF1A, and analyzed the resulting mixture by gel filtration chromatography (Figure 7B). Remarkably, we found that sNASP, H3, H4 and ASF1A all eluted in a stable complex, comparable to that seen when using pre-folded H3–H4 dimers to reconstitute the complex (Figure 2C), in addition to a small quantity of aggregates eluting in the void volume of the column (Figure 7B).

Whilst reconstituting the sNASP–H3–H4–ASF1A from unfolded histones, we noticed that the order in which the histones and chaperones were combined, greatly affected the efficiency of reconstitution, with some orders of addition producing marked precipitation over others. We reasoned that some interactions may need to occur before others in order to drive complex assembly. We investigated this possibility by systematically combining the four components (sNASP, H3, H4 and ASF1A) in all possible orders of addition, and measured the reconstitution efficiency by quantifying the precipitate formed (Figure 7C). Interestingly, when we ranked the orders of addition according to total relative precipitate, we see that in the top quartile of most efficient reconstitutions, sNASP is always added before ASF1A. Conversely, we see that in the bottom quartile (the least efficient reconstitutions), ASF1A is always added before sNASP. This reveals a general rule in which efficient reconstitution of the tetrameric complex *in vitro* requires sNASP to be added before ASF1A. These results support the idea that sNASP functions upstream of ASF1A in the histone chaperoning pathway.

To test if the H3 and H4 are folded correctly with the reconstituted sNASP–H3–H4–ASF1A complex, we carried out a tetrasome reconstitution assay (42,45), with the view that a correctly folded H3–H4 dimer should behave identically to a prefolded H3–H4 dimer in both its efficiency of tetrasome reconstitution and its migration pattern during native PAGE. In this assay, the ability of a chaperone to mitigate aggregation and promote correct folding of a tetrasome particle is assessed under increasing histone to DNA ratios. At lower ratios, disomes are formed (a single H3–H4 dimer associated with DNA), leading to tetrasome formation (two H3–H4 dimers associated with DNA) and then aggregation when histones are in a large excess, as has been observed previously (42,44). Similarly, we see that both sNASP and ASF1A can individually aid in depositing H3–H4 onto DNA, with sNASP preferentially forming tetrasomes and ASF1A preferentially forming disomes, whereas traditional salt dialysis results in tetrasome formation (Supplementary Figure S4). Furthermore, when sNASP and ASF1A are combined, both disomes and tetrasomes are formed with disomes predominating (Supplementary Figure S4).

Direct addition of pre-folded H3–H4 dimers to DNA results in poor assembly of tetrasomes, with increasing histone to DNA ratios resulting in aggregation (Figure 7E, lanes 1–3). The addition of unfolded H3 and H4 results in even poorer tetrasome reconstitution, as would be expected (Figure 7E, lanes 4–6). However, when H3–H4 dimers are first assembled in an sNASP–H3–H4–ASF1A complex before adding DNA, reconstitution is greatly enhanced (Figure 7E, lanes 7–9), as may be expected from the chaperones preventing non-productive thermodynamic traps, thereby guiding more efficient tetrasome reconstitution. Importantly, however, there is no observable difference in either the efficiency of tetrasome reconstitution or the migration pattern of histone–DNA complexes when comparing sNASP–H3–H4–ASF1A complex formed from pre-folded H3–H4 dimers (Figure 7E, lanes 7–9) and sNASP–H3–H4-–ASF1A complex formed from monomeric H3 and H4 (Figure 7E, lanes 10–12). This suggests that sNASP and ASF1A are fully capable of efficiently folding an H3–H4 dimer from unfolded monomeric substrates *in vitro* and supports the notion that sNASP functions upstream of ASF1A in the histone chaperoning pathway.

## DISCUSSION

sNASP represents a family of TPR motif containing chaperones that specifically interact with histones H3 and H4. *In vivo* sNASP has been isolated in complex with other co-chaperones, including ASF1A/B, RbAp46 and the histone acetyl-transferase HAT1, which represents the major soluble source of H3–H4 within the cell. Previously, we identified that the H3 binding site of sNASP overlaps significantly with that of ASF1A (25), suggesting that (1) binding site competition may be important in H3–H4 maturation, and (2), as ASF1A and sNASP exist in complex with each other, either sNASP or ASF1A contain secondary interaction sites with their histone substrate.

In this current study, we have presented a detailed biochemical investigation into how sNASP and ASF1A co-chaperone histones H3 and H4, and present a model in which sNASP and ASF1A cooperate through both competitive and compatible interactions to fold and retain an H3–H4 dimer in an aggregation-resistant state (Figure 7F). In this model, formation of a sNASP–H3 complex is upstream of the sNASP–H3–H4–ASF1A complex. Importantly, in a cellular context this is most likely also associated with the HAT1-complex and other accessory factors (10,11,19,20). Interaction between sNASP and H3 in the sNASP–H3 complex is predominantly mediated by the high affinity TPR-peptide interaction (25). However, displacement of both mb1 and mb13 monobody probes also suggested that secondary modes of interaction between sNASP and H3 are present, involving both the acidic domain and the outer surface of the TPR domain/capping region (Figure 6B). In contrast to sNASP, ASF1A mediates its interaction predominantly with the folded surface of the H3–H4 dimer (30,32), agreeing with our observation that ASF1A is unable to compete with sNASP for binding of H3 in the absence of H4 (Figure 1C–G). Upon H3 folding with H4, sequestration of residues important in mediating the TPR–H3 peptide interaction (Ala127, Arg131) (Figure 1A) within the histone fold may act to reduce the grip of sNASP on this region and result in a transition from sNASP being bound at the C-terminus of H3 to ASF1A being bound as the H3–H4 dimer forms. sNASP, however, is still retained within the ASF1–H3–H4 complex through its secondary interaction sites involving the central acidic domain and the outer surface of the TPR/capping region (Figures 5 and 6). Due to their ability to solubilise the otherwise aggregation prone ASF1–H3–H4 complex, these secondary modes of interaction between sNASP and H3–H4 most likely aid in preventing non-specific interactions within a cellular context, ensuring that a soluble pool of histones is maintained at all times. In this regard, the molecular model we propose is consistent with findings showing that NASP contributes to the fine-tuning of a soluble reservoir of H3–H4 through inhibition of chaperone-mediated autophagy (CMA) (21). Furthermore, as sNASP and ASF1A are sufficient for folding of an H3–H4 dimer *in vitro*, the isolation of protein folding chaperones associated with histone H3 (17) may relate to a role of the these proteins in CMA/quality control, rather than in generating an H3–H4 dimer.

Most biochemical analysis to date has involved individual analysis of histone chaperone function in isolation, this being especially true for NASP (15,16,54,55). However, H3 and H4 are found in multi-chaperone complexes in the cell, and so the individual function of each chaperone is likely only to play an important but small role in the histone deposition process. We have shown that sNASP has synergistic functions with ASF1 towards H3 and H3–H4. Interestingly, H4 has also been shown to have specific chaperones, binding RbAp46 and HAT1, which form a stable dimeric HAT1 complex, important in acetylation of lysine 5 and 12 of H4 (26,56,57). A recent crystal structure of the *Saccharomyces cerevisiae* homolog of the RbAp46–HAT1 dimer bound to H4 and H3 peptides revealed that nearly half of the H4 molecule is sequestered within the binding pockets of the HAT1 complex (27), including the majority of the N-terminal tail and the α1 helix of the histone core domain. Interestingly, the α1 helix of H4 and the α3 helix of H3 reside in close proximity to each other on the same side of the histone fold dimer. Binding and releasing of these regions within the core histone fold domain may therefore aid in guiding H3 and H4 down a productive folding pathway.

The role that sNASP plays in the folding, maturation and storage of histones is complex and most likely involves a number of other chaperones in addition to ASF1. In order to further understand the process, high-resolution structures of the stable components of the pathway, namely the sNASP–H3 dimer and the sNASP–H3–H4–ASF1A tetramer, need to be solved and the dynamic intermediates probed through complementary, biophysical means. The role of additional factors, such as the HAT1 holo-enyme, and how they function in concert with sNASP and ASF1 also has to be addressed. Importantly, novel approaches will need to be developed in order to observe the rapid process of ‘histone hand-off’ *in vivo* and to validate such mechanisms, as we and others have proposed (17,21). In this report, we have biochemically characterized the sNASP–H3 and sNASP–H3–H4–ASF1A complexes and demonstrated that multiple interaction interfaces exist between sNASP and histones. We have shown how these contend and cooperate with the chaperone ASF1A to form a folded H3–H4 dimer, establishing a mechanistic platform for the further investigation of these dynamic histone chaperoning processes.
